# Dataset of the effect of difficulty messaging on academic cheating in middle school Chinese children

**DOI:** 10.1016/j.dib.2022.108405

**Published:** 2022-06-20

**Authors:** Li Zhao, Junjie Peng, Liyuzhi D. Dong, Yaxin Li, Haiying Mao, Brian J. Compton, Jiacheng Ye, Guoqiang Li, Gail D. Heyman, Kang Lee

**Affiliations:** a Department of Psychology, Hangzhou Normal University, No. 2318 Yuhangtang Rd, Yuhang District, Hangzhou 311121, PR China; b Department of Primary Education, Hangzhou Normal University, PR China; c Dr Eric Jackman Institute of Child Study, University of Toronto, 45 Walmer Rd., Toronto, Ontario M5R 2X2, Canada; d Department of Psychology, University of California San Diego, USA

**Keywords:** Academic cheating, Academic integrity, Academic honesty, Academic dishonesty, Difficulty messaging, Moral psychology, Development, Middle school children

## Abstract

The present dataset was reported in a paper entitled “Effects of test difficulty messaging on academic cheating among middle school children” [1]. It reports the findings of an experimental study that used a naturalistic math test-taking paradigm to assess children's academic cheating behavior under different test difficulty messaging conditions. The participants were Grade 8 middle school children (*N* = 201). The primary dependent measures were whether each participant spontaneously decided to cheat (*presence of cheating*), and among participants who cheated, the specific number of test items on which they cheated (*extent of cheating*). We used logistic regression, ANOVA, and Pearson correlation to assess whether various predictor variables (e.g., conditions) predicted the presence of cheating or the extent of cheating. This dataset should be of interest to researchers who are interested in the development of moral behavior in children generally, and academic dishonesty in particular.

**Specifications Table**

**Table:** 

Subject	Psychology
Specific subject area	Developmental and Educational Psychology
Type of data	Tables and figures
How data were acquired	Experiment with naturalistic paradigm
Data format	Raw and analyzed statistical data
Parameters for data collection	Participants were recruited from six randomly selected Grade 8 classes at a large, full-day middle school in Eastern China, and parents or legal guardians provided informed consent for them to participate. Three children did not participate in one of the parts of the experiment and thus were removed from the dataset.
Description of data collection(Max 400 characters.)	Children were given a math test and informed of its difficulty level (described as either *grade level, easy* or *hard*) even though the actual difficulty level was the same for all three conditions. Unbeknownst to the children, we took photos of their test sheets after they turned them in. Two weeks later, we returned the test sheets to the children for them to self-score based on an answer key that we provided. We then compared children's answer sheets before and after the self-scoring procedure to determine whether they had falsely inflated their score during the self-scoring procedure (*presence of cheating*) and for children who cheated, the number of items on which they cheated (*extent of cheating*).
Data source location	City/Town/Region: Middle school located in eastern China.Country: China
Data accessibility	Repository name: Mendeley dataDirect URL to data: https://doi.org/10.17632/f3chzwykxm.1
Related research article	Zhao, L., Peng, J., Dong, L. D., Li, Y., Mao, H., Compton, B. J., Ye, J., Li, G., Heyman, G. D., & Lee, K. (2022). Effects of test difficulty messaging on academic cheatingamong middle school children. *Journal of Experimental Child Psychology, 220*, 105417–105431. https://doi.org/10.1016/j.jecp.2022.105417

**Value of the Data**

• This dataset provides information about how many middle school participants from eastern China cheated on a math test, and among the children who chose to cheat, the extent of their cheating.
• This dataset presents information about key predictor variables related to children's cheating behavior.
• This dataset can be used as a source for researchers who are interested in studying the development of moral behavior in general and academic cheating in particular.
• This dataset can be used as a source for researchers who are interested in the effect of messages provided by teachers regarding children's academic cheating behavior.
• This dataset can be used as a source for researchers who are interested in understanding children's moral behavior in a naturalistic experimental setting.

## 1 Data Description

The dataset is based on an experimental study with 201 students at a middle school in eastern China. We measured the impact of test difficulty messaging on cheating behavior through the use of a naturalistic math test situation, and collected behavioral data [2]. The math test consisted of 20 multiple choice items and ten fill-in-the-blank items. The final test items are listed in the https://doi.org/10.1016/j.jecp.2022.105417. According to our prior survey of the teachers from the mathematics department and some of the junior second-year students, the school has never recommended or required students to buy and read these extracurricular tutoring books. None of the students interviewed purchased themselves and thus the likelihood that the participants had read this book and done the exercises before the experiment was extremely small.

We looked at children's actual scores on the math test (Table 1), the cheating rate for all children (Table 2), the cheating extent for the children who cheated (Table 3), and correlations among study variables (Table 4) to better understand the cheating behavior of middle school children.

**Table 1:** Actual test scores for all children.

Messaging Condition	*M*	*SD*
Grade level (*n* = 66)	46.14	15.66
Easy (*n* = 66)	45.35	15.78
Hard (*n* = 69)	46.77	14.47
Total (*n* = 201)	46.09	15.23

**Table 2:** Cheating rate for all children.

Messaging Condition	Percent Cheating
Grade level (*n* = 66)	40.9% (27/66)
Easy (*n* = 66)	62.1% (41/66)
Hard (*n* = 69)	58.0% (40/69)
Total (*n* = 201)	53.7% (108/201)

*Note.* Frequencies are shown in parentheses.

**Table 3:** Cheating extent for the children who cheated.

Messaging Condition	*M*	*SD*	*Range*
Grade level (*n* = 27)	10.63	9.02	2 ∼ 34
Easy (*n* = 41)	16.29	13.45	1 ∼ 61
Hard (*n* = 40)	8.20	5.59	2 ∼ 24
Total (*n* = 108)	11.88	10.57	1 ∼ 61

**Table 4:** Correlations between study variables.

			Actual test score	Presence of cheating	Cheating extent	Gender
Grade level(*n* = 66)	Actual test score	*r*(*p*)	\	\	\	\
	Presence of cheating	*r*(*p*)	-0.150(0.229)	\	\	\
	Cheating extent	*r*(*p*)	-0.094(0.455)	0.678(0.000)	\	\
	Gender	*r*(*p*)	0.052(0.680)	0.092(0.460)	0.073(0.561)	\

Easy(*n* = 66)	Actual test score	*r*(*p*)	\	\	\	\
	Presence of cheating	*r*(*p*)	-0.100(0.423)	\	\	\
	Cheating extent	*r*(*p*)	-0.002(0.989)	0.603(0.000)	\	\
	Gender	*r*(*p*)	-0.039(0.755)	0.062(0.621)	-0.041(0.745)	\

Hard(*n* = 69)	Actual test score	*r*(*p*)	\	\	\	\
	Presence of cheating	*r*(*p*)	0.174(0.152)	\	\	\
	Cheating extent	*r*(*p*)	-0.014(0.911)	0.693(0.000)	\	\
	Gender	*r*(*p*)	-0.183(0.132)	0.026(0.829)	0.039(0.749)	\

*Note.* Spearman r values are shown outside the parentheses and *p* values are in the parentheses.

## 2 Experimental Design, Materials and Methods

The primary data for this study was collected via naturalistic mathematical tests that we designed. The dataset includes demographic information, children's actual test scores, children's cheating rates, correlations between study variables, how children cheated, and how much children cheated.

The data came from 204 eighth-graders at a middle school in eastern China. Of these, 107 were boys (52.45%) and 97 were girls. Using a cluster sampling method, six classes were randomly selected from all eighth grade classes at the public middle school where the study took place. Two of these classes were randomly assigned to each of three experimental conditions. Of the total 204 participants mentioned above, three of them (one in each condition) were excluded because they took the first stage of the math test but failed to participate in the second self-scoring phase due to illness or other reasons. The final valid data was 201, of which 107 were boys (53.23%) and 94 were girls, with 66 children in the grade level condition (33 boys), 66 children in the easy condition (37 boys) and 69 children in the hard condition (37 boys). Gender data on all students was collected before the testing began. Because this was a real test, it would be unusual for us to ask children to report their ages on the test sheet. We obtained the students’ age information afterwards from the school administration: mean age = 13.36 years, *SD* = 0.49. In addition, 32 Grade 8 students from a different class were chosen to participate in a pretest to confirm that the experimental materials were appropriate for children of this age.

Data collection took places in the children's classroom and was divided into three phases: a testing phase, an experimenter scoring phase, and a student self-scoring phase. During the testing phase the experimenter gave instructions and handed out test sheets. The experimenter informed each class of the difficulty condition of the test before they began working on it. In the experimenter scoring stage, which took place after the 20-min test period was over, the experimenter retrieved the test sheets and left the classroom. Unbeknownst to the children, the experimenter took a photo of each child's test sheet, which was later used to calculate the child's actual score (see Table 1). The experimenter then returned each child's test sheet, and during the self-scoring phase, children were instructed to score their test sheet by marking it according to an answer key provided the experimenter. The experimenter then retrieved the self-scored test sheets. Children's actual test scores were compared to their self-scored test scores, which were coded as cheating = 1 if there was a difference between the actual score and the self-scored score, and no cheating = 0 if there was no such difference, as shown in Table 2. In addition, the difference between the two scores were coded as cheating extent.

Among the children who cheated, we identified four different strategies children used to improve their test scores. Altering responses to multiple choice questions only, code = 1; altering responses to fill-in-the-blank questions only, code = 2; altering both types of responses, code = 3; reporting an inflated final score without altering any responses, code = 4.

## Ethics Statement

The authors obtained informed consent from children's parents or legal guardians to participate in the study. Children's participation was voluntary and they could withdraw from the study at any time. To protect children's privacy, we only reported children's actual test scores to their home room teacher. No teachers at the school were able to find out which children cheated during the self-scoring phase. This study was approved by the Review Committee of the Center for Cognitive and Brain Disease Research of Hangzhou Normal University (Ref. 20,201,221). Data collection was carried out in accordance with research ethics guidelines and regulations. The authors have the right to share the anonymized dataset publicly.

## CRediT authorship contribution statement

**Li Zhao:** Conceptualization, Methodology, Supervision, Writing – review & editing. **Junjie Peng:** Methodology, Data curation, Software, Writing – original draft. **Liyuzhi D. Dong:** Methodology, Writing – original draft. **Yaxin Li:** Methodology, Data curation. **Haiying Mao:** Methodology, Data curation, Software. **Brian J. Compton:** Supervision, Writing – review & editing. **Jiacheng Ye:** Investigation, Data curation. **Guoqiang Li:** Supervision, Resources. **Gail D. Heyman:** Supervision, Writing – review & editing. **Kang Lee:** Methodology, Supervision, Writing – review & editing.

## Declaration of Competing Interest

The authors declare that they have no known competing financial interests or personal relationships that could have appeared to affect the work reported in this paper.

## Appendix Supplementary materials

Image, application 1
